# Supplementary material for: Vinorelbine Improves the Efficacy of Sorafenib against Hepatocellular Carcinoma: A Promising Therapeutic Approach
Source: Int J Mol Sci. 2024 Jan 26;25(3):1563. doi: 10.3390/ijms25031563 (PMC10855313; doi:10.3390/ijms25031563)
Supplement: Supplementary file 1 [file ijms-25-01563-s001.zip › ijms-2813614-supplementary.pdf]

Supplementary Materials

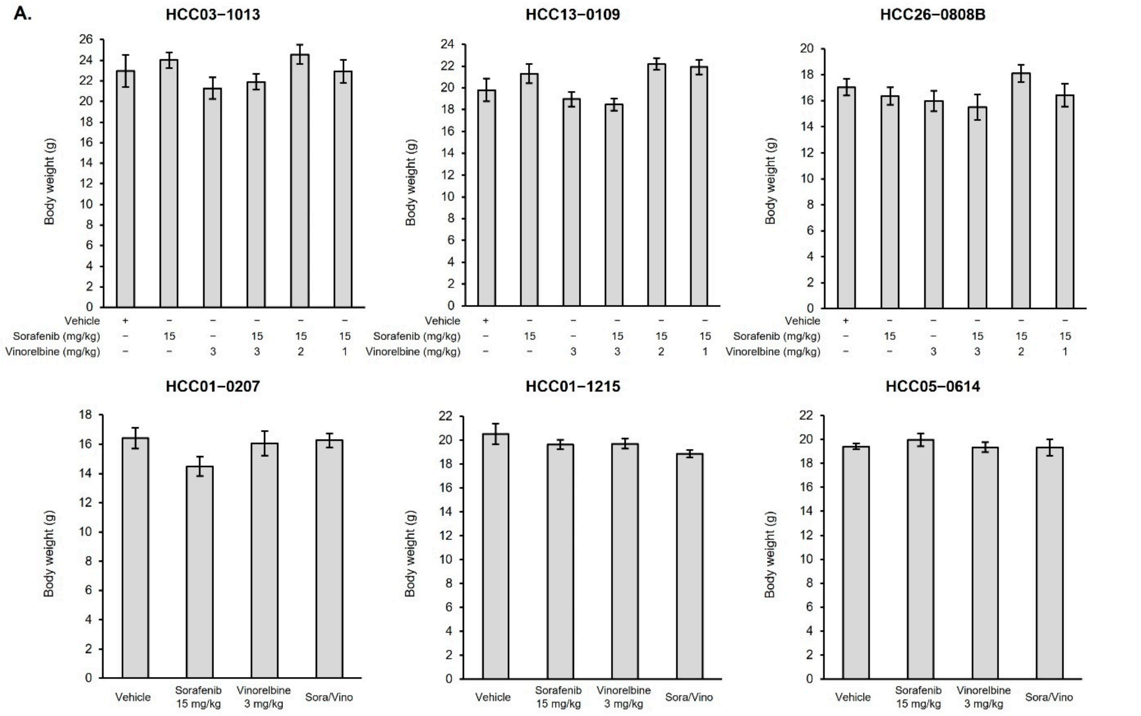

Supplementary Figure S1A. Effects of sorafenib, vinorelbine, and Sora/Vino on the body weights of HCC PDX models. Mice bearing the indicated HCC tumors were treated with the vehicle, sorafenib, vinorelbine, or Sora/Vino, as indicated. The body weight of the treated mice was measured, and the mean body weight ± SEs at the time of sacrifice were presented.

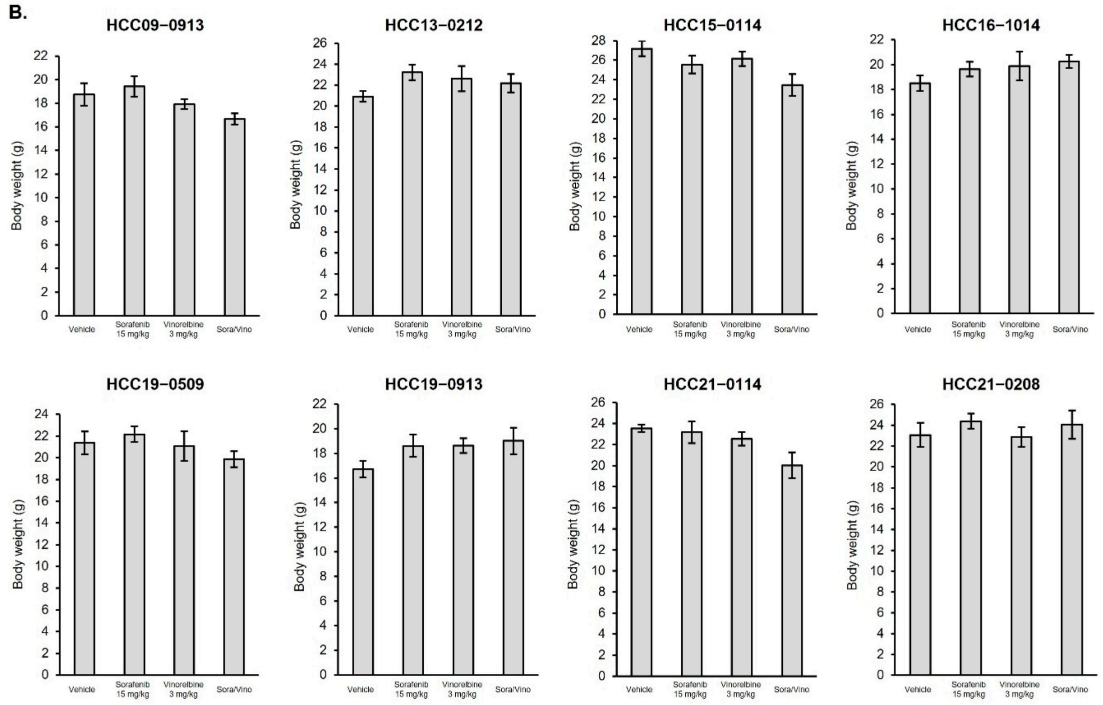

Supplementary Figure S1B. Effects of sorafenib, vinorelbine, and Sora/Vino on the body weights of HCC PDX models. Mice bearing the indicated HCC tumors were treated with the vehicle, sorafenib, vinorelbine, or Sora/Vino,

as indicated. The body weight of the treated mice was measured, and the mean body weight ± SEs at the time of sacrifice were presented.

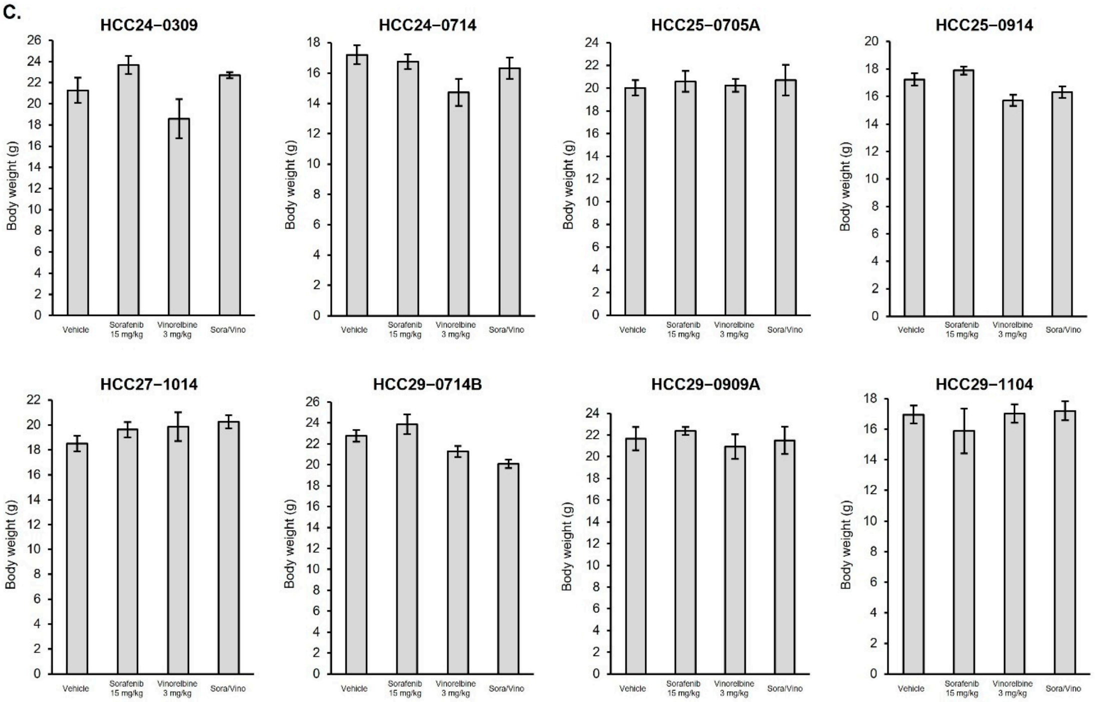

Supplementary Figure S1C. Effects of sorafenib, vinorelbine, and Sora/Vino on the body weights of HCC PDX models. Mice bearing the indicated HCC tumors were treated with the vehicle, sorafenib, vinorelbine, or Sora/Vino, as indicated. The body weight of the treated mice was measured, and the mean body weight ± SEs at the time of sacrifice were presented.

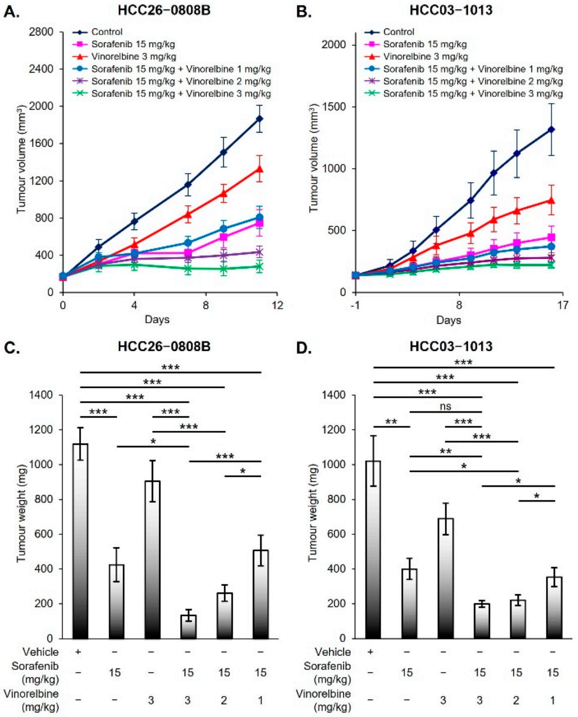

Supplementary Figure S2. Dose-dependent effects of vinorelbine and Sora/Vino in the HCC26−0808B and HCC03−1013 PDX models. Mice bearing the indicated tumors were treated with the vehicle plus PBS, sorafenib,

vinorelbine, or Sora/Vino, as indicated. (A, B) The mean tumor volumes ± SEs; and (C, D) the mean corresponding tumor weights ± SEs at sacrifice are shown (* $p < 0.05$; ** $p < 0.01$; *** $p < 0.001$; ns, no significance; ANOVA followed by Tukey's test).

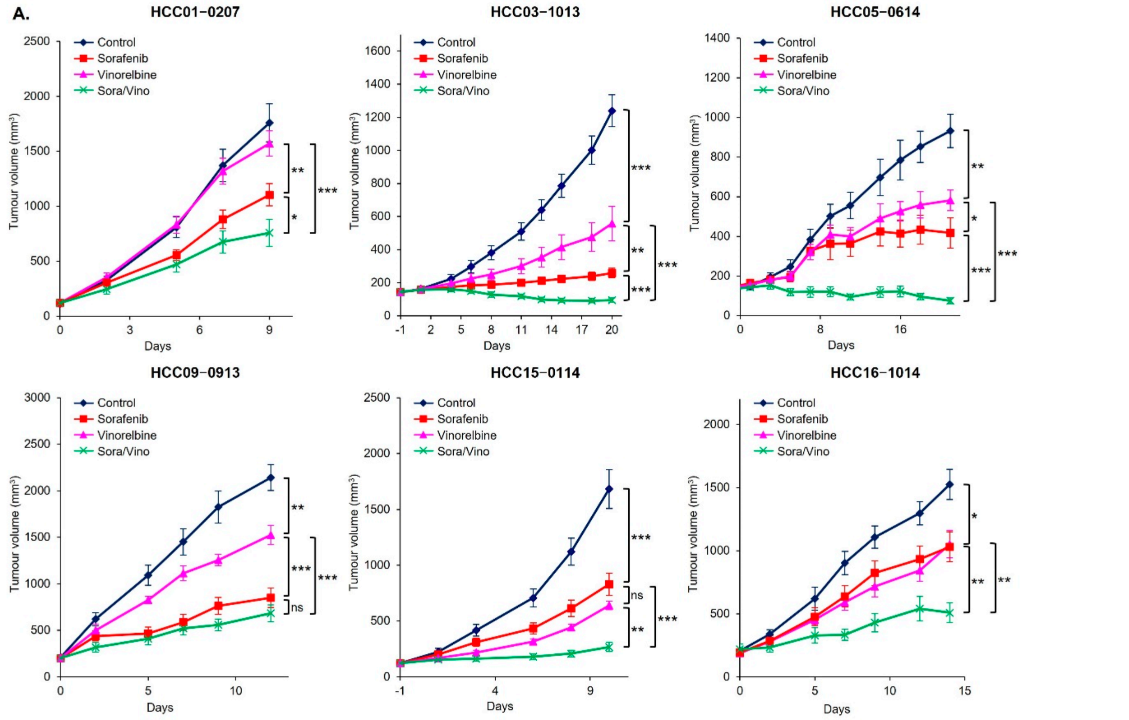

Supplementary Figure S3A. Enhanced antitumor effects of the combination of vinorelbine with sorafenib. Mice bearing HCC tumors were treated with the vehicle, sorafenib, vinorelbine, and Sora/Vino over a specific treatment period, as described in Section 4. The mean tumor volumes ± SEs are plotted (* $p < 0.05$; ** $p < 0.01$; *** $p < 0.001$; ns, no significance; ANOVA followed by Tukey's test). Notably, Sora/Vino demonstrated a significant reduction in tumor growth compared to the vehicle or monotherapy groups.

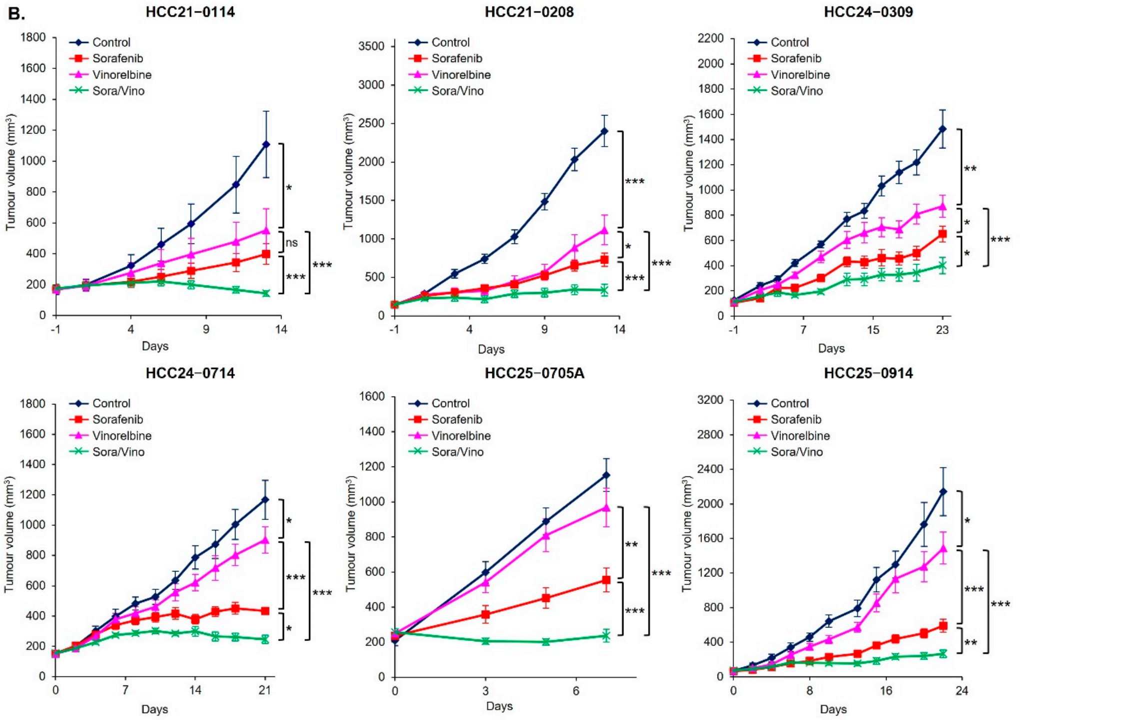

Supplementary Figure S3B. Enhanced antitumor effects of the combination of vinorelbine with sorafenib. Mice bearing HCC tumors were treated with the vehicle, sorafenib, vinorelbine, or Sora/Vino over a specific treatment period, as described in Section 4. The mean tumor volumes ± SEs are plotted (* $p < 0.05$; ** $p < 0.01$; *** $p < 0.001$; ns, no significance; ANOVA followed by Tukey's test). Notably, Sora/Vino demonstrated a significant reduction in tumor growth compared to the vehicle or monotherapy groups.

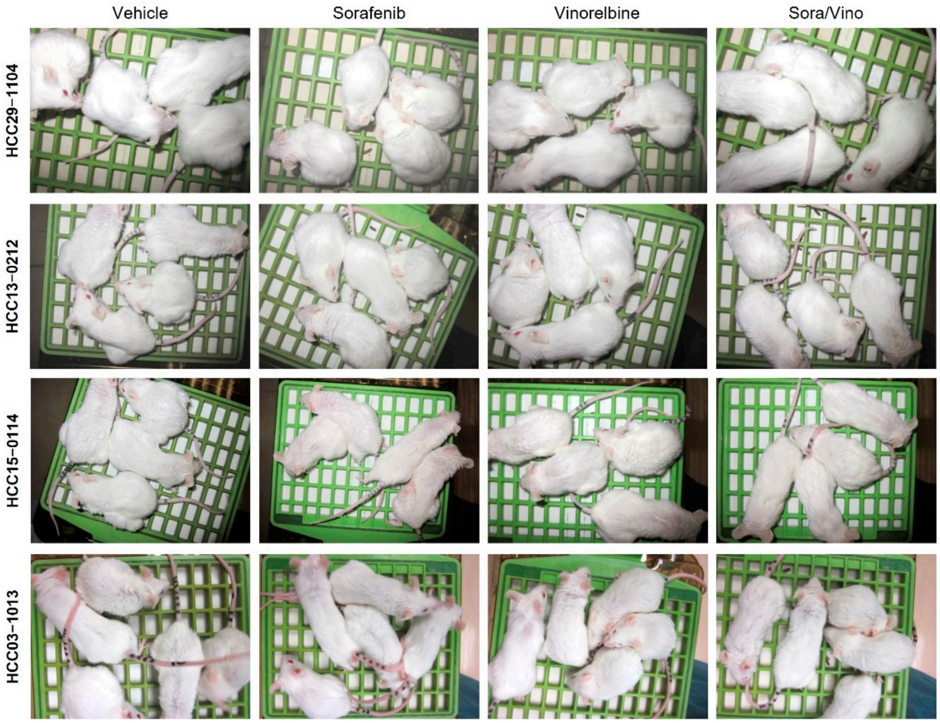

Supplementary Figure S4. Minimal drug toxicity and side effects of Sora/Vino in HCC PDX models. Representative photographs of the HCC PDX models treated with the vehicle, sorafenib, vinorelbine, or Sora/Vino revealed that all experimental mice displayed a healthy coat, normal food and water intake, typical social interactions and activity levels, and no signs of aggression among cage mates. Their active movement suggests that the administered dosage, drug toxicity, and side effects posed minimal risks to the treated mice.

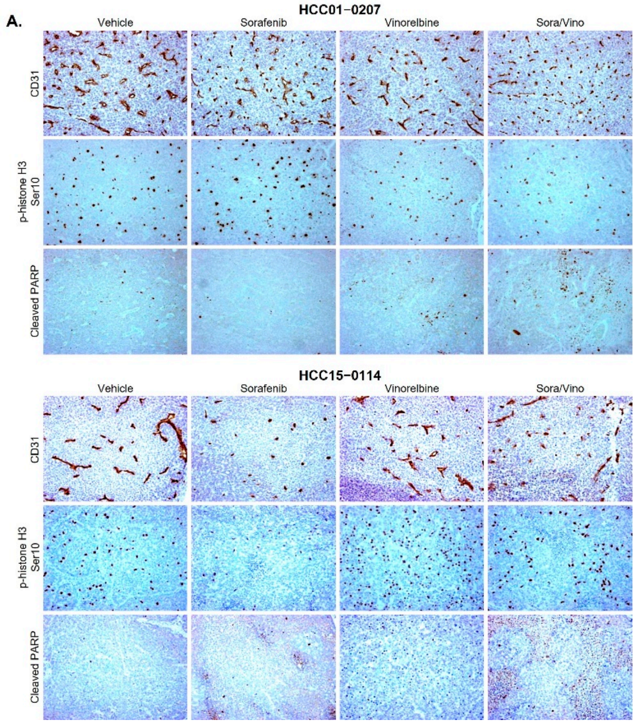

Supplementary Figure S5A. Sora/Vino treatment inhibited angiogenesis and induced apoptosis in the HCC01–0207 and HCC15–0114 PDX models. Mice bearing the indicated tumors were randomly divided into four groups and treated with the vehicle, sorafenib, vinorelbine, or Sora/Vino. Harvested tumors were processed for IHC, as described in Section 4. Representative images of tumor sections from vehicle- and drug-treated mice stained for CD31 (blood vessels), p-histone H3 Ser10, and cleaved PARP are shown. Sorafenib treatment reduced the number and size of blood vessels, vinorelbine treatment led to a significant increase in p-histone H3 Ser10-positive cells, and Sora/Vino treatment resulted in a significant increase in cleaved PARP-positive cells. Tumor necrosis was also observed in the stained sections of the HCC PDX models.

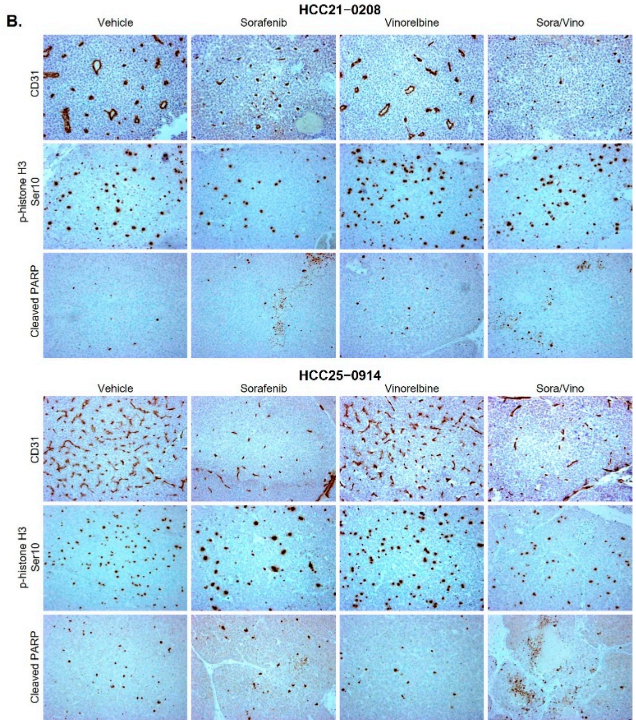

Supplementary Figure S5B. Sora/Vino treatment inhibited angiogenesis and induced apoptosis in the HCC21−0208 and HCC25−0914 PDX models. Mice bearing the indicated tumors were randomly divided into four groups and treated with the vehicle, sorafenib, vinorelbine, or Sora/Vino. Harvested tumors were processed for IHC, as described in Section 4. Representative images of tumor sections from vehicle- and drug-treated mice stained for CD31 (blood vessels), p-histone H3 Ser10, and cleaved PARP are shown. Sorafenib treatment reduced the number and size of blood vessels, vinorelbine treatment led to a significant increase in p-histone H3 Ser10-positive cells, and Sora/Vino treatment resulted in a significant increase in cleaved PARP-positive cells. Tumor necrosis was also observed in the stained sections of the HCC PDX models.

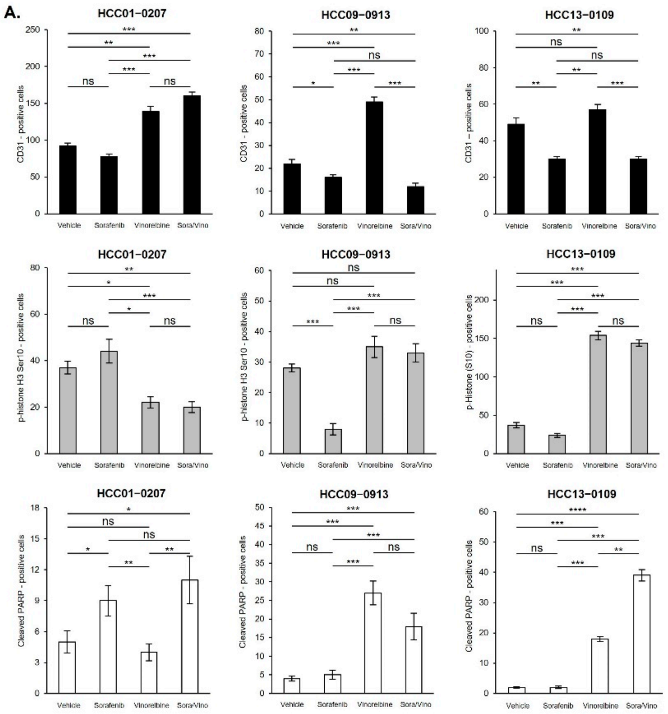

Supplementary Figure S6A. Quantification analysis of CD31-, p-histone H3 Ser10-, and cleaved PARP-positive cells in the HCC01−0207, HCC09−0913, and HCC13−0109 PDX models treated with the vehicle, sorafenib, vinorelbine, and Sora/Vino. Data are presented as the mean ± SEs (* $p < 0.05$; ** $p < 0.01$; *** $p < 0.001$; ns, no significance; Student's t-test).

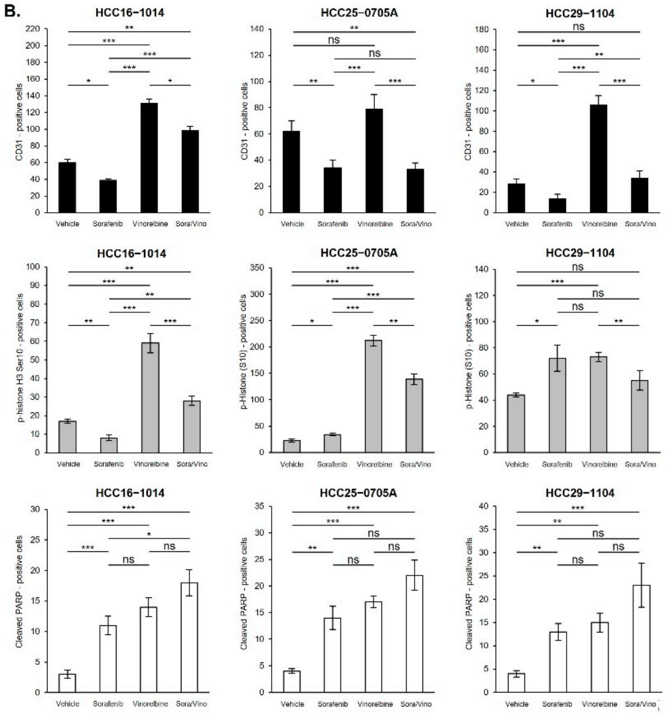

Supplementary Figure S6B. Quantification analysis of CD31-, p-histone H3 Ser10-, and cleaved PARP-positive cells in the HCC16−1014, HCC25−0705A, and HCC29−1104 PDX models treated with the vehicle, sorafenib, vinorelbine, and Sora/Vino. Data are presented as the mean ± SEs (* $p < 0.05$; ** $p < 0.01$; *** $p < 0.001$; ns, no significance; Student's t-test).

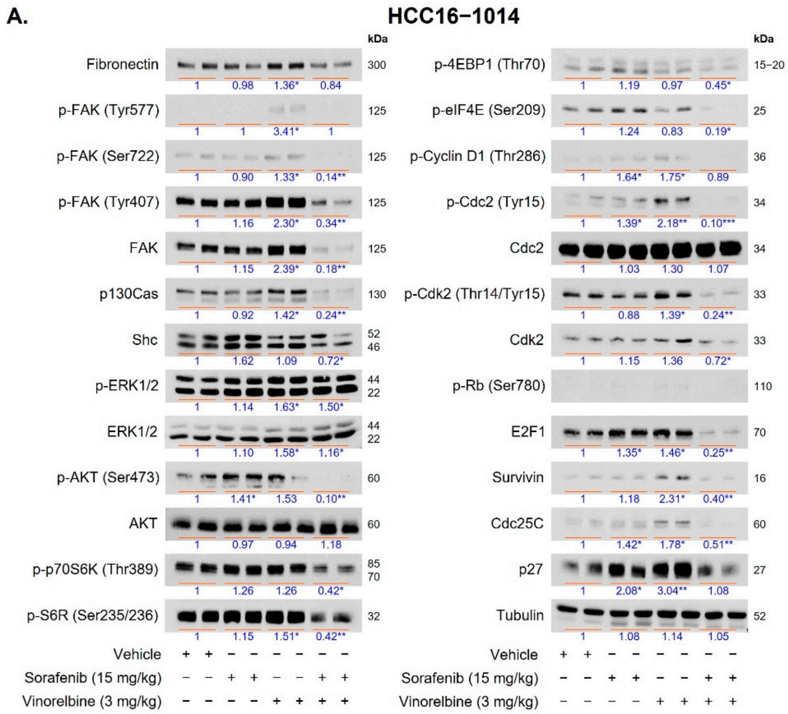

Supplementary Figure S7A. Sora/Vino synergistic effects in the treatment of HCC PDX models. Mice bearing HCC16−1014 tumors were treated with sorafenib, vinorelbine, or Sora/Vino following the methods described in Section 4. Tumors (n = 10/group) from each treatment group were harvested, pooled and snap frozen. Two pooled tumors from each treatment group were subjected to western blot analysis with the indicated antibodies. The total density of each corresponding protein band was quantified, normalized to tubulin (loading control), and expressed

as a fold change relative to the vehicle group. A value greater (or lesser) than 1 indicated that the expression level of the protein of interest was greater (or lower) than that in the control group Statistical analysis was performed using ANOVA followed by Tukey's test (* $p < 0.05$; ** $p < 0.01$; *** $p < 0.001$; ns, no significance). Western blot analysis revealed that HCC16−1014 tumors treated with Sora/Vino exhibited a synergistic effect in reducing the protein levels of p-FAK (Ser722), p-FAK (Tyr407), FAK, p-Cyclin D1 (Thr286), p-Cdc2 (Tyr15), p-Cdk2 (Thr14/Tyr15), Cdk2, p-AKT (Ser473), E2F1, p130Cas, Cdc25C, p70S6K (Thr389), p-S6R (Ser235/236), and p-eIF4E (Ser209) compared to the vehicle or monotherapies.

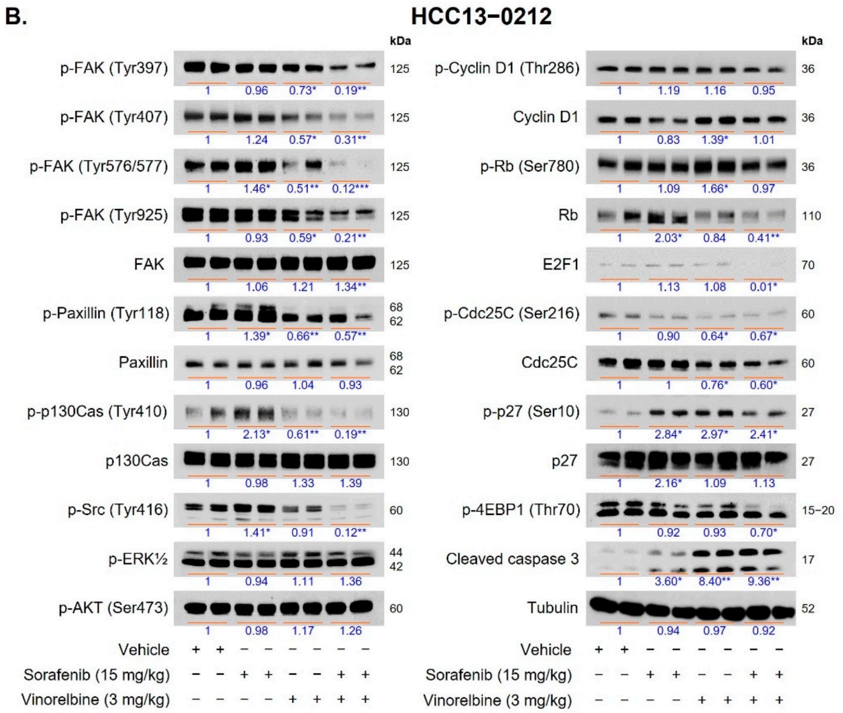

Supplementary Figure S7B. Sora/Vino synergistic effects in the treatment of HCC PDX models. Mice bearing HCC13−0212 tumors were treated with sorafenib, vinorelbine, or Sora/Vino following the methods described in Section 4. Tumors (n = 10/group) from each treatment group were harvested, pooled and snap frozen. Two pooled tumors from each treatment group were subjected to western blot analysis with the indicated antibodies. The total density of each corresponding protein band was quantified, normalized to tubulin (loading control), and expressed as a fold change relative to the vehicle group. A value greater (or lesser) than 1 indicated that the expression level of the protein of interest was greater (or lower) than that in the control group Statistical analysis was performed using ANOVA followed by Tukey's test (* $p < 0.05$; ** $p < 0.01$; *** $p < 0.001$; ns, no significance). Western blot analysis revealed that HCC13−0212 tumors treated with Sora/Vino exhibited a synergistic effect in reducing the protein levels of p-FAK (Tyr397), p-FAK (Tyr407), p-FAK (Tyr576/577), p-FAK (Tyr925), Rb, p-p130Cas (Tyr410), E2F1, p-paxillin (Tyr118), p-Cdc25C (Ser216), Cdc25C, and p-Src (Tyr416) compared to the vehicle or monotherapy groups.

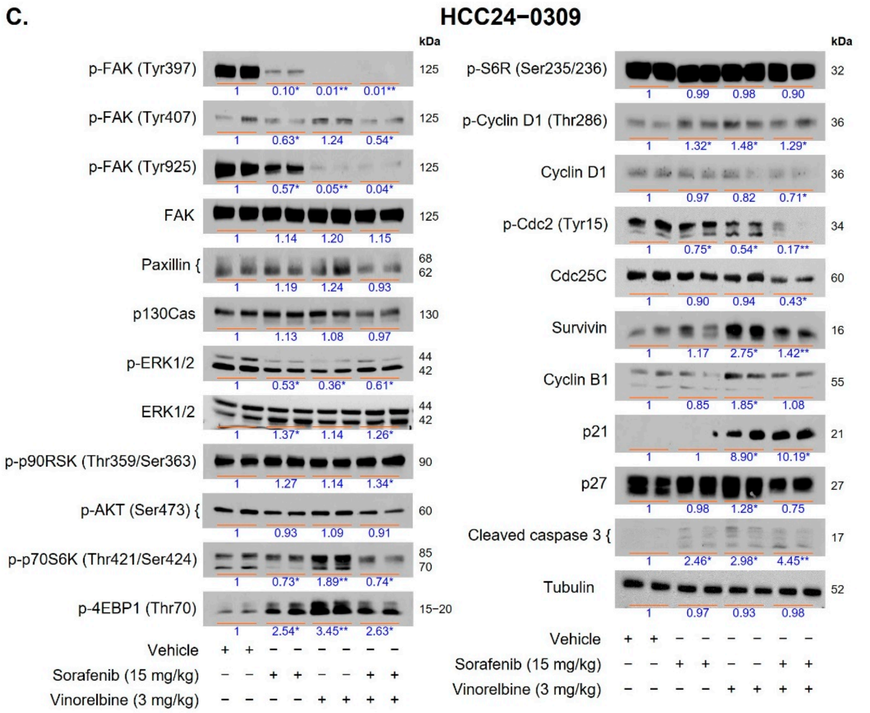

Supplementary Figure S7C. Sora/Vino synergistic effects in the treatment of HCC PDX models. Mice bearing HCC24−0309 tumors were treated with sorafenib, vinorelbine, or Sora/Vino following the methods described in Section 4. Tumors (n = 10/group) from each treatment group were harvested, pooled and snap frozen. Two pooled tumors from each treatment group were subjected to western blot analysis with the indicated antibodies. The total density of each corresponding protein band was quantified, normalized to tubulin (loading control), and expressed as a fold change relative to the vehicle group. A value greater (or lesser) than 1 indicated that the expression level of the protein of interest was greater (or lower) than that in the control group Statistical analysis was performed using ANOVA followed by Tukey's test (* $p < 0.05$; ** $p < 0.01$; *** $p < 0.001$; ns, no significance). Western blot analysis revealed that HCC24−0309 tumors treated with Sora/Vino exhibited a synergistic effect in reducing the protein levels of p-FAK (Tyr397), p-FAK (Tyr925), p-Cdc2 (Tyr15), Cdc25C, and p130Cas compared to the vehicle or monotherapy groups.

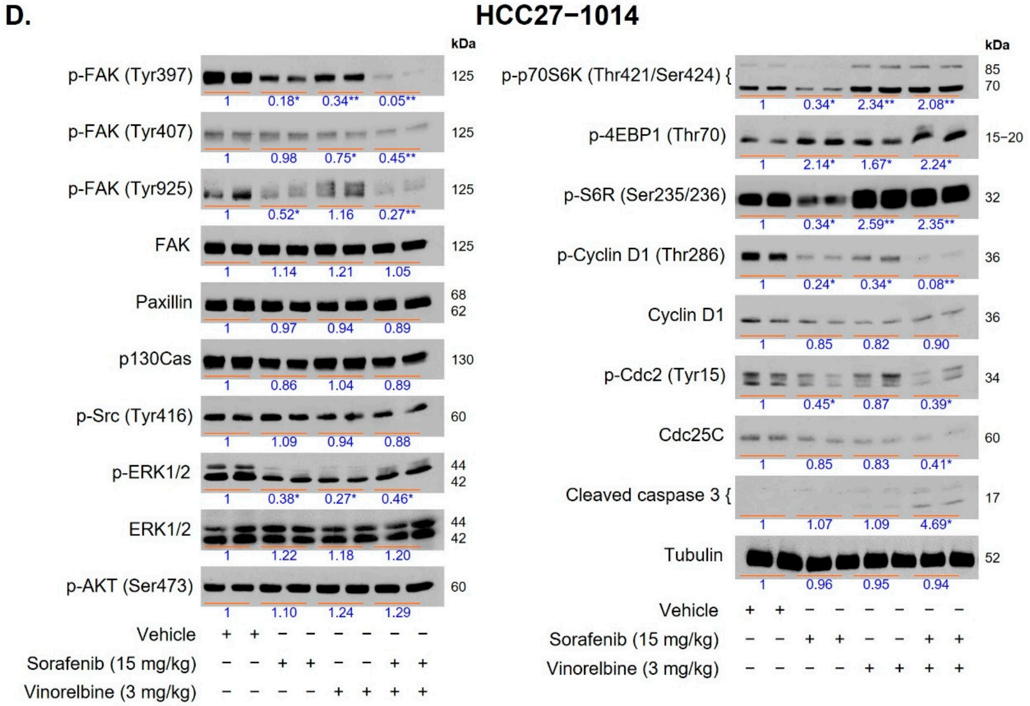

Supplementary Figure S7D. Sora/Vino synergistic effects in the treatment of HCC PDX models. Mice bearing HCC27−1014 tumors were treated with sorafenib, vinorelbine, or Sora/Vino following the methods described in Section 4. Tumors (n = 10/group) from each treatment group were harvested, pooled and snap frozen. Two pooled tumors from each treatment group were subjected to western blot analysis with the indicated antibodies. The total density of each corresponding protein band was quantified, normalized to tubulin (loading control), and expressed as a fold change relative to the vehicle group. A value greater (or lesser) than 1 indicated that the expression level of the protein of interest was greater (or lower) than that in the control group Statistical analysis was performed using ANOVA followed by Tukey's test (* $p < 0.05$; ** $p < 0.01$; *** $p < 0.001$; ns, no significance). Western blot analysis revealed that HCC27−1014 tumors treated with Sora/Vino exhibited a synergistic effect in reducing the protein levels of p-FAK (Tyr397), p-FAK (Tyr407), p-FAK (Tyr925), p-Cyclin D1 (Thr286), Cyclin D1, p-Cdc2 (Tyr15), and p-ERK1/2 compared to the vehicle or monotherapy groups.

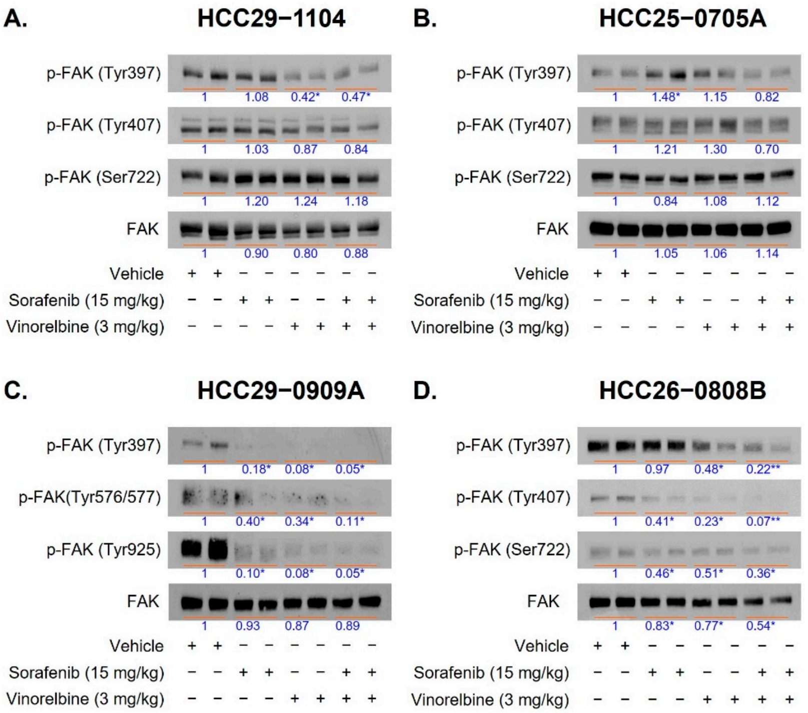

Supplementary Figure S8. Consistently reduced FAK phosphorylation by Sora/Vino in HCC PDX models. Mice bearing the indicated tumors were treated with sorafenib, vinorelbine, or Sora/Vino, as described in Section 4. Harvested tumors were subjected to Western blot analysis using the indicated antibodies, as described in Section 4. Treatment with Sora/Vino in (A) HCC29−1104, (B) HCC25−0705A, (C) HCC29−0909A, and (D) HCC26−0808B significantly reduced the levels of phosphorylated FAK at Tyr397, Tyr407, and Tyr925 compared to the vehicle or monotherapy groups.

Supplementary Table S1. Sensitivity of HCC PDX models to Sora/Vino treatment. A total of 22 HCC PDX models were treated with the vehicle, sorafenib, vinorelbine, or Sora/Vino. The T/C ratio of each treatment was calculated by comparison to the vehicle group. Tumors with T/C ratios < 0.3 were considered sensitive, those with T/C ratios between 0.3 and 0.42 were considered moderately sensitive, and those with T/C ratios > 0.42 were considered less sensitive (resistant). Approximately 86.4% (19/22) of HCC models were considered sensitive, whereas 13.6% (3/22) were considered moderately sensitive when treated with Sora/Vino.

HCC PDX models	Treatments and T/C Ratio				
	Vehicle	Sorafenib 15mg/kg	Vinorelbine 3mg/kg	Sora/Vino	Sorafenib *vs* Sora/Vino *p*-values
HCC01-0207	1	0.532	0.961	0.4037	0.1195
HCC01-1215	1	0.5776	0.6397	0.1629	1.418×10^{-5}
HCC03-1013	1	0.194	0.3338	0.0816	7.776×10^{-4}
HCC05-0614	1	0.3294	0.5143	0.0732	2.193×10^{-3}
HCC09-0913	1	0.4512	0.9035	0.3098	0.0337
HCC13-0109	1	0.2323	0.1473	0.0204	1.959×10^{-5}
HCC13-0212	1	0.1592	0.1503	0.0297	6.844×10^{-9}
HCC15-0114	1	0.3246	0.6021	0.1506	5.269×10^{-3}
HCC16-1014	1	0.581	0.5837	0.3382	6.532×10^{-3}
HCC19-0509	1	0.386	0.324	0.0886	2.688×10^{-5}
HCC19-0913	1	0.2628	0.3253	0.05	1.224×10^{-4}
HCC21-0114	1	0.343	0.4352	0.1231	6.418×10^{-5}

HCC21-0208	1	0.2622	0.4207	0.1116	3.541×10^{-4}
HCC24-0309	1	0.348	0.487	0.201	0.0187
HCC24-0714	1	0.3097	0.7768	0.1994	2.885×10^{-4}
HCC25-0705A	1	0.4395	0.7869	0.152	6.688×10^{-4}
HCC25-0914	1	0.2639	0.8243	0.1102	1.175×10^{-3}
HCC26-0808B	1	0.3806	0.8103	0.1206	0.0106
HCC27-1014	1	0.2999	0.4152	0.1235	3.230×10^{-4}
HCC29-0714B	1	0.3839	0.5791	0.1461	2.653×10^{-3}
HCC29-0909A	1	0.3058	0.5981	0.1386	7.889×10^{-5}
HCC29-1104	1	0.3375	0.6262	0.1652	1.218×10^{-3}

Supplementary Table S2. List of primary antibodies used in Western blot analysis.

No.	Antibody	Target Protein	Brand	Cat. No.	Host
1	p-4EBP1 (Thr70)	Phosphorylated eukaryotic translation initiation factor 4E binding protein 1 at Thr 70	Cell Signaling Technology	#13396	Rabbit
2	AKT	AKT Serine/Threonine Kinase	Cell Signaling Technology	#9272	Rabbit
3	p-AKT (Ser473)	Phosphorylated AKT Serine/Threonine Kinase at Ser 473	Cell Signaling Technology	#9271	Rabbit
4	Bcl-xL	BCL2 like 1	Cell Signaling Technology	#2764	Rabbit
5	c-myc	Transcriptional regulator Myc-like	Proteintech	10057-1-AP	Rabbit
6	p-c-myc (Ser62)	Phosphorylated transcriptional regulator Myc-like at Ser 62	Cell Signaling Technology	#13748	Rabbit
7	CDC25C	Cell Division Cycle 25C	Cell Signaling Technology	#4688	Rabbit
8	p-CDC25C (Ser216)	Phosphorylated Cell Division Cycle 25C at Ser 216	Cell Signaling Technology	#4901	Rabbit
9	Cdc2	Cyclin Dependent Kinase 1	Santa Cruz Biotechnology	sc-954	Rabbit
10	p-Cdc2 (Tyr15)	Phosphorylated Cell Dependent Kinase 1 at Tyr 15	Cell Signaling Technology	#9111	Rabbit
11	Cdk2	Cyclin Dependent Kinase 2	Cell Signaling Technology	#2546	Rabbit
12	p-Cdk2 (Thr14/Tyr15)	Phosphorylated Cyclin Dependent Kinase 2 at Thr 14/Tyr 15	Santa Cruz Biotechnology	sc-28435-R	Rabbit
13	Cleaved Caspase 3	Activated caspase-3 cleaved at Asp 175	Cell Signaling Technology	#9661	Rabbit
14	Cyclin A2	Cyclin A2	Cell Signaling Technology	#4656	Mouse
15	Cyclin B1	Cyclin B1	Cell Signaling Technology	#4138	Rabbit
16	Cyclin D1	Cyclin D1	Cell Signaling Technology	#2978	Rabbit
17	p-Cyclin D1 (Thr286)	Phosphorylated Cyclin D1 at Thr 286	Cell Signaling Technology	#3300	Rabbit
18	E2F1	E2F transcription factor 1	Santa Cruz Biotechnology	sc-251	Mouse
19	E-cadherin	Cadherin 1,	Proteintech	20874-1-AP	Rabbit
20	eIF4E	Eukaryotic translation initiation factor 4E	Cell Signaling Technology	#9742	Rabbit
21	p-eIF4E (Ser209)	Phosphorylated Eukaryotic translation initiation factor 4E at Ser 209	Cell Signaling Technology	#9741	Rabbit
22	ERK1/2	p44 and p42 MAP Kinase (Erk1 and Erk2)	Santa Cruz Biotechnology	sc-94	Rabbit
23	p-ERK1/2	Phosphorylated p44 and p42 MAP Kinase (Erk1 and Erk2) at Thr202 and Tyr204	Cell Signaling Technology	#4370	Rabbit
24	FAK	Focal adhesion kinase	Cell Signaling Technology	#13009	Rabbit
25	p-FAK (Tyr397)	Phosphorylated Focal adhesion kinase at Tyr 397	Cell Signaling Technology	#8556	Rabbit
26	p-FAK (Tyr407)	Phosphorylated Focal adhesion kinase at Tyr 407	Millipore	07-829	Rabbit

27	p-FAK (Tyr576/577)	Phosphorylated Focal adhesion kinase at Tyr 576/577	Cell Signaling Technology	#3281	Rabbit
28	p-FAK (Tyr925)	Phosphorylated Focal adhesion kinase at Tyr 925	Cell Signaling Technology	#3284	Rabbit
29	Fibronectin	Fibronectin 1	CalBiochem	CP13L	Mouse
30	p-mTOR (Ser2448)	Phosphorylated mammalian target of rapamycin (mTOR) kinase at Ser 2448	Cell Signaling Technology	#5536	Rabbit
31	p130Cas	p130 Crk-associated substrate (Cas)	Santa Cruz Biotechnology	sc-20029	Mouse
32	p-p130Cas (Tyr410)	Phosphorylated p130 Crk-associated substrate (Cas) at Tyr 410	Cell Signaling Technology	#4011	Rabbit
33	P21	p21 Waf1/Cip1 family of cyclin dependent kinase inhibitor 1A	Cell Signaling Technology	#2947	Rabbit
34	P27	Cip/Kip family of cyclin dependent kinase inhibitor 1B	Cell Signaling Technology	#3686	Rabbit
35	p-p27 (Ser10)	Phosphorylated Cip/Kip family of cyclin dependent kinase inhibitor 1B at Ser 10	Abcam	ab62364	Rabbit
36	Paxillin	Paxillin	Cell Signaling Technology	#12065	Rabbit
37	p-Paxillin (Tyr118)	Phosphorylated paxillin at Tyr 118	Cell Signaling Technology	#2541	Rabbit
38	p-p70S6k (Thr389)	Phosphorylated ribosomal protein S6 kinase B1 at Thr 389	Cell Signaling Technology	#9205	Rabbit
39	p-p70S6k (Thr421/Ser424)	Phosphorylated ribosomal protein S6 kinase B1 at Thr 421/Ser 424	Cell Signaling Technology	#9204	Rabbit
40	p-p90RSK (Thr359/Ser363)	Phosphorylated ribosomal protein S6 kinase A1/A2 at Thr 359/Ser 363	Cell Signaling Technology	#9344	Rabbit
41	Rb	Retinoblastoma (Rb) transcriptional corepressor 1	Cell Signaling Technology	#9313	Rabbit
42	p-Rb (Ser780)	Phosphorylated Rb at Ser 780	Cell Signaling Technology	#8180	Rabbit
43	p-S6R (Ser235/236)	Phosphorylated ribosomal protein S6 at Ser 235/236	Cell Signaling Technology	#4858	Rabbit
44	Shc	SHC-transforming protein 1	Cell Signaling Technology	#2432	Rabbit
45	p-Shc (Tyr239/240)	Phosphorylated SHC-transforming protein 1 at Tyr239/240	Cell Signaling Technology	#2434	Rabbit
46	p-Src (Tyr416)	Phosphorylated Src family tyrosine kinase at Tyr 416	Cell Signaling Technology	#2101	Rabbit
47	Survivin	Survivin	Cell Signaling Technology	#2808	Rabbit
